# Bibliography

**Published:** 1901-09-15

**Authors:** 


### BIBLIOGRAPHY.

The seventh edition of *Dental Medicine*, by Dr. F. J. S. Gorgas, has just been issued from the press of P. Blakiston's Son & Co. This edition has been enlarged and much of the matter re-edited. It contains some thirty more pages than the former edition, and the type used is smaller, so that there is a considerable increase in matter. Many of the newer dental remedies are considered, and important additions have been made to the therapeutic section. We are pleased to note that an effort has been made to more systematically arrange the matter into a classification that shall make the work more useful as a class text book. We have never been quite satisfied with this book as a text work, because of its arrangement. It no doubt serves the purpose of a practitioner's hand book better than any work of its kind, but not as a text book for school purposes. It should take up the discussion of dental diseases and their diagnosis first, and then classify them for therapeutic purposes; and, when they have been studied—not from their pathological characteristics wholly, but with their therapeutic treatment in view—the remedies should be studied in therapeutic classes. The alphabetical arrangement of the remedies might then be added, together with such pharmaceutical helps as may be desirable for prescribing, compounding and dispensing. This, it seems to us, would make an ideal text book and would be none the less helpful to the practitioner. We have never thought much of the plan of attaching formulæ to each article, except where they may be of value in illustrating the therapeutic application of the drug under discussion, and we think much of the space so used in this work could be more advantageously utilized.

As a combination of text book for students and hand book for practitioners, this work has held its place for a long time, and is likely to continue to do so, at least until the time comes when our pharmacology shall have been worked out to the extent of giving us a treatise on that subject and our therapeutic practice becomes sufficiently uniform to enable us to dogmatize; then we can have our treatise and hand book or text book in separate volumes. The work is neatly printed, and the author and publishers are to be commended for their efforts to keep this branch of our art abreast of the times.

